# Flame-Retardant Polyvinyl Alcohol Materials: Mechanisms, Design Strategies, and Multifunctional Applications

**DOI:** 10.3390/polym17192649

**Published:** 2025-09-30

**Authors:** Dehui Jia, Lulu Xu, Danni Pan, Yi Xiao, Yan Zhang, Yao Yuan, Wei Wang

**Affiliations:** 1 Fujian Provincial Key Laboratory of Functional Materials and Applications, School of Materials Science and Engineering, Xiamen University of Technology, Xiamen 361024, China; j_dehui@163.com (D.J.); p_danni@163.com (D.P.); 2020000004@xmut.edu.cn (Y.X.); 2 School of Chemical Engineering, University of New South Wales, Sydney, NSW 2052, Australia; 3 Zhejiang Provincial Engineering Research Center for Green and Low-Carbon Dyeing & Finishing, Ministry of Education, Zhejiang Sci-Tech University, Hangzhou 310018, China; zy52360@zstu.edu.cn; 4 School of Engineering, RMIT University, Melbourne, VIC 3001, Australia; wei.wang@rmit.edu.au

**Keywords:** polyvinyl alcohol, flame retardancy, smoke toxicity, modification, flame retardant mechanism

## Abstract

Polyvinyl alcohol (PVA), a water-soluble, biodegradable, and biocompatible polymer, has garnered significant attention in recent years for its applications such as packaging, electronics, biomedical materials, and water treatment. However, its high flammability poses a substantial limitation in fire-sensitive environments. To address this challenge, significant research efforts have been devoted to improving the flame retardancy and suppressing the smoke toxicity of PVA through various strategies. This review presents diverse modification strategies that have been developed for PVA, including physical blending with polymers and nanofillers, chemical modifications such as esterification, acetalization, and crosslinking, and advanced surface engineering techniques such as plasma treatment, layer-by-layer assembly, and surface grafting. Beyond fire safety, these modifications enable multifunctional applications, expanding PVA use in optical, energy, sensing, and biomedical fields. Finally, this review explores current challenges, environmental considerations, and future directions for the development of sustainable, high-performance flame-retardant PVA systems.

## 1. Introduction

PVA is a versatile synthetic polymer produced through the hydrolysis of poly(vinyl acetate), featuring a linear backbone densely populated with hydroxyl groups [1,2,3,4,5,6]. This highly polar structure facilitates extensive hydrogen bonding, endowing PVA with exceptional water solubility, strong film-forming capability, oil and grease resistance, and compatibility with a wide range of fillers and additives [7,8,9,10,11]. PVA-based films exhibit excellent mechanical strength, outstanding barrier performance, and good biocompatibility, making them suitable for diverse applications such as adhesives [12,13,14], packaging films [15,16,17], textile finishing agents [18,19], construction binders [20,21,22], antimicrobial materials [23,24], flexible optoelectronic devices [25,26], and gas barrier coatings [27,28]. Despite these advantages, PVA displays poor fire resistance [29]. Upon exposure to heat, the polymer undergoes rapid thermal degradation and produces large amounts of combustible volatiles such as acetaldehyde, acetic acid, and hydrocarbons. This process accelerates flame propagation, generates high heat release rates, and produces dense smoke and toxic products, all of which significantly limit its use in fire sensitive environments. Consequently, the development of efficient flame-retardant strategies for PVA is essential to broaden its range of safe and practical uses.

Efforts to enhance the flame retardancy of PVA have concentrated on both chemical and physical modification pathways [30,31]. Recent research has introduced a variety of strategies, including the use of halogen-free flame retardants, the incorporation of inorganic nanofillers, the development of synergistic phosphorus–nitrogen systems, and the construction of bio-based flame-retardant networks. These approaches not only mitigate ignition propensity and reduce heat release but also influence char layer formation, limit smoke generation, and lower the emission of toxic gases, thereby promoting environmentally sustainable solutions [32,33]. In addition, the latest advancements in PVA flame-retardant design increasingly emphasize multifunctionality, where fire resistance is coupled with added features such as antimicrobial activity [34,35,36], ultraviolet protection [37,38,39], enhanced barrier performance, or mechanical reinforcement [40,41]. This multifunctional approach is enabling PVA to meet the demands of emerging applications in areas such as wearable electronics, sustainable packaging, biomedical devices, and high-performance protective coatings. By integrating these capabilities, PVA is evolving from a conventional polymer into a versatile material platform capable of addressing both safety requirements and advanced functional needs in next-generation technologies.

Considering the hydroxyl-rich structural characteristics of PVA, various modification strategies can be employed to enhance its performance [42,43]. The abundance of hydroxyl groups provides numerous reactive sites that can participate in hydrogen bonding [44,45], covalent grafting [46,47], or coordination interactions, enabling the introduction of flame-retardant components with improved compatibility and uniform dispersion within the polymer matrix. By carefully tailoring the molecular interactions, it is possible to construct a dynamic cross-linking network in which multiple hydrogen bonds act as both physical junctions and reversible sacrificial bonds, thereby imparting enhanced structural integrity under normal conditions and enabling energy dissipation during thermal or mechanical stress [48,49,50]. Furthermore, these reactive sites can be utilized to incorporate functional moieties such as conductive fillers, antibacterial agents, or stimuli-responsive groups, endowing PVA with additional capabilities including electrical conductivity, antimicrobial activity, and environmental responsiveness. Such structural modifications not only expand the functional scope of PVA but also allow the creation of advanced multifunctional materials suitable for demanding applications in fields such as flame-retardant engineering, flexible electronics, biomedical devices, and smart packaging.

Bio-based flame retardants have emerged as a promising class of additives for PVA, offering both environmental benefits and functional enhancements [51,52,53,54,55,56]. Derived from renewable resources such as lignin [57,58], chitosan [33,59], starch [60,61], and phytic acid [62,63,64], these compounds possess intrinsic flame-retardant elements including phosphorus, nitrogen, and aromatic structures that facilitate the formation of stable char layers, limit heat release, and improve thermal stability during combustion [65]. Their incorporation into PVA matrices opens avenues for developing eco-friendly materials that maintain high mechanical integrity while delivering effective fire protection, along with potential secondary functions such as antimicrobial activity, electromagnetic interference shielding [66], UV-blocking capability [67], thermal conductivity enhancement [68,69], and ultra-long fire alarm [70]. The chemical structures of these bio-based flame retardants provide multiple reactive sites that can interact with the hydroxyl-rich backbone of PVA, yet achieving uniform dispersion and strong interfacial bonding is often hindered by differences in polarity, hydrophilicity, or particle agglomeration. Overcoming these barriers requires tailored molecular design, surface modification of bio-based additives, and advanced processing techniques to ensure long-term stability, consistent flame-retardant performance, and compatibility with diverse end-use requirements.

While several reviews have addressed the flame retardancy of polymers, including PVA, most have centered on combustion behavior and traditional bulk modifications, leaving a gap in connecting the unique molecular characteristics of PVA with emerging strategies and multifunctionality. This review fills that gap by systematically examining the principles of fire resistance and the latest advances in PVA design. It discusses chemical modifications such as esterification, acetalization, and crosslinking to improve thermal stability and char formation, as well as physical blending with polymers and nanofillers, where hydrogen bonding, electrostatic interactions, and van der Waals forces enhance dispersion and interfacial adhesion. Advanced surface engineering methods, including plasma treatment, layer-by-layer assembly, and grafting, are also highlighted for improving surface-specific properties while maintaining bulk integrity. Beyond flame retardancy, the review emphasizes multifunctionality, showing how bio-based strategies and surface engineering enable PVA to acquire additional features such as antimicrobial activity, sensing, and energy harvesting. By integrating these perspectives, the work proposes a design principle that shifts focus from fire safety alone to multifunctionality, offering a framework for developing sustainable, high-performance PVA materials for advanced applications in biomedical, energy, and sensing fields.

## 2. Flame-Retarding Mechanisms for PVA

### 2.1. The Combustion Properties of PVA

**Figure 1:**
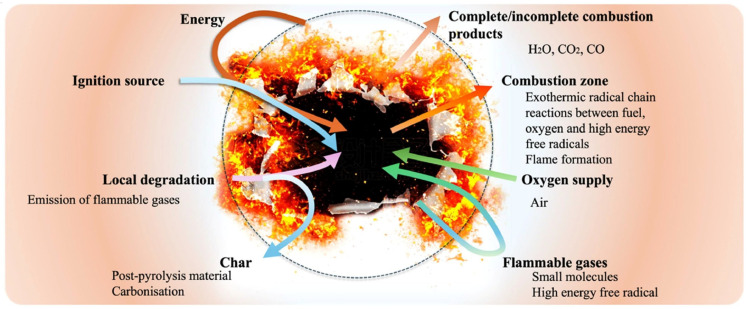
Combusting cycle of a typical incendiary material [74]. Copyright 2025. Reproduced with permission from American Chemical Society.

The combustion behavior of PVA is strongly influenced by its thermal decomposition and the structure of its polymer chains [71]. Under a nitrogen atmosphere, pure PVA undergoes thermal degradation in distinct stages that govern its flammability and heat release characteristics. The first stage, occurring between approximately 227 and 300 °C, involves the removal of side hydroxyl groups. This process results in the loss of about 30% of the polymer mass and produces major pyrolysis products including water, isolated and conjugated polyenes, aldehydes, and ketones. In addition to generating flammable volatiles, this stage initiates the formation of early char fragments that affect subsequent decomposition [72]. The second stage, occurring between 300 and 450 °C, is dominated by random chain scission and cyclization reactions along the polymer backbone [73]. These reactions further degrade the main chains, release additional combustible gases, and form a carbon-rich residue [74]. When exposed to sufficient heat, the material undergoes decomposition followed by combustion, with the underlying mechanism illustrated in Figure 1. The interplay of these decomposition stages contributes to high flammability, rapid heat release, and dense smoke production, emphasizing the importance of developing effective flame-retardant strategies to enhance the fire safety of PVA materials.

### 2.2. The Mechanism of Flame Retardants in PVA

The flame-retardant behavior of PVA arises from a synergistic combination of gas-phase and condensed-phase processes that act in concert to suppress combustion and enhance thermal stability. This dual mechanism enables the material to resist ignition, slow thermal degradation, and maintain structural integrity under high-temperature conditions, highlighting the effectiveness of carefully engineered flame-retardant strategies in improving the safety and performance of PVA-based systems.

#### 2.2.1. Gas-Phase Flame Retardant Mechanism

The gas-phase flame-retardant mechanism of PVA involves the suppression of combustion through both the release of non-combustible gases and the quenching of reactive radicals [75]. When exposed to heat, flame-retardant additives, especially those containing phosphorus or nitrogen, decompose to release gases such as water, carbon dioxide, and ammonia. These gases dilute flammable volatiles and lower the local oxygen concentration within the flame zone, effectively reducing the flammability and slowing the combustion process. In addition to gas dilution, certain additives generate phosphorus- or nitrogen-centered radicals that capture highly reactive species, including hydrogen and hydroxyl radicals, which are responsible for propagating flame reactions. This radical scavenging interrupts the chain reactions of combustion, decreasing flame spread and heat release. The gas-phase mechanism is particularly important in controlling early-stage pyrolysis and volatile emissions, and it often works in synergy with condensed-phase processes such as char formation.

#### 2.2.2. Condensed-Phase Flame Retardant Mechanism

The condensed-phase flame-retardant mechanism involves the formation of a thermally stable char layer that serves as a protective barrier during combustion [76,77,78]. Upon heating, flame-retardant additives, especially those containing phosphorus, nitrogen, or derived from bio-based sources, facilitate dehydration and cross-linking reactions within the PVA matrix. These reactions result in a dense carbonaceous residue on the polymer surface, which provides multiple protective functions. The char layer acts as a physical shield, limiting heat transfer to the underlying polymer, slowing thermal degradation, and reducing the emission of combustible volatiles [79]. Certain intumescent additives further enhance this effect by causing the char to expand into a foam-like structure, which improves insulation and further restricts heat penetration. In addition, inorganic fillers or layered nanomaterials can catalyze the formation of the char, strengthen its structural integrity, and create tortuous pathways that hinder the diffusion of pyrolysis gases, enhancing the barrier effect. Collectively, these condensed-phase processes decrease flame propagation, lower heat release rates, and suppress smoke production. When combined with gas-phase mechanisms, the condensed-phase action significantly enhances the overall fire resistance of PVA-based materials while maintaining mechanical performance and compatibility with multifunctional additive systems.

#### 2.2.3. Synergistic Flame-Retardant Mechanism

The synergistic flame-retardant mechanism of PVA arises from the combined action of multiple flame-retardant pathways, including gas-phase inhibition, condensed-phase char formation, and catalytic or intumescent effects. In this mechanism, different types of flame-retardant additives are strategically integrated to complement each other, resulting in enhanced fire protection compared with single-component systems [80]. The interplay of these mechanisms reduces flame propagation, lowers heat release rates, and suppresses smoke and toxic gas emission more effectively than individual additives alone. By exploiting synergistic interactions, flame-retardant PVA can achieve high-performance fire resistance while retaining multifunctional properties, making it suitable for advanced applications in sustainable and safe polymeric materials.

## 3. Design Strategies for Flame-Retardant PVA

### 3.1. Physical Blending Modification of PVA

Physical blending modification of PVA is a widely applied strategy to improve its performance by incorporating other polymers, fillers, or functional additives without altering its primary chemical structure [81]. Through physical blending, properties such as mechanical strength, flexibility, water resistance, and thermal stability can be effectively tuned, while additional functionalities such as flame retardancy, conductivity, or antimicrobial activity may also be introduced. Compared with chemical modification, physical blending offers the advantages of simplicity, cost-effectiveness, and scalability, making it a practical route for tailoring PVA-based materials to meet the demands of a wide range of applications. Table 1 provides a comparative overview of different material systems, highlighting their compositions, modification strategies, and corresponding flame-retardant properties.

#### 3.1.1. Hydrogen Bond Association

**Figure 2:**
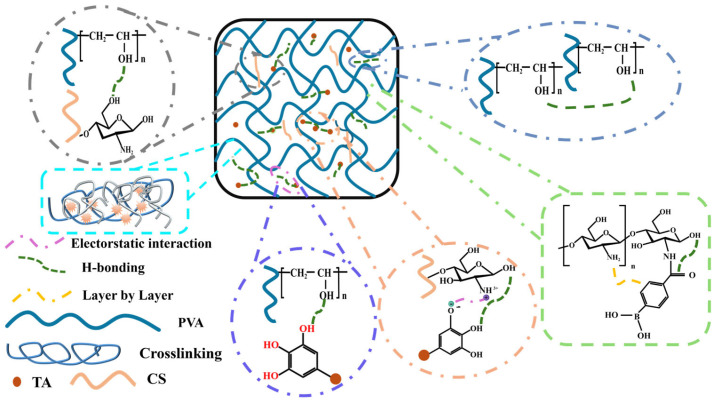
Schematic illustration of structural modifications and interaction mechanisms in flame-retardant PVA-based systems.

Hydrogen bonding is a key mechanism in the physical blending of PVA due to its hydroxyl-rich backbone [9]. The hydroxyl groups along the PVA chains act as active sites that readily form intermolecular hydrogen bonds with polymers, small molecules, or inorganic fillers introduced during blending. Figure 2 presents a schematic illustration of structural modifications and interaction mechanisms in flame-retardant PVA-based systems, including hydrogen-bond associations. These non-covalent interactions improve compatibility, enabling uniform dispersion and stable composite formation without covalent modification. In blends with polymers such as starch, chitosan, or cellulose derivatives, hydrogen bonding enhances miscibility, reduces phase separation, and strengthens interfacial adhesion. Regeneration studies on PVA have gained increasing attention because they assess the recyclability and long-term stability of the material, both of which are essential for sustainable polymer design. The water solubility, reversible hydrogen bonding, and semicrystalline structure of PVA allow it to be reprocessed through dissolution and recasting or thermal cycling while largely maintaining structural integrity. Similarly, when combined with inorganic fillers like layered silicates, metal oxides, or carbon-based nanomaterials, hydrogen bonds reinforce filler–matrix interactions, leading to enhanced barrier properties, flame retardancy, and thermal stability.

**Figure 3:**
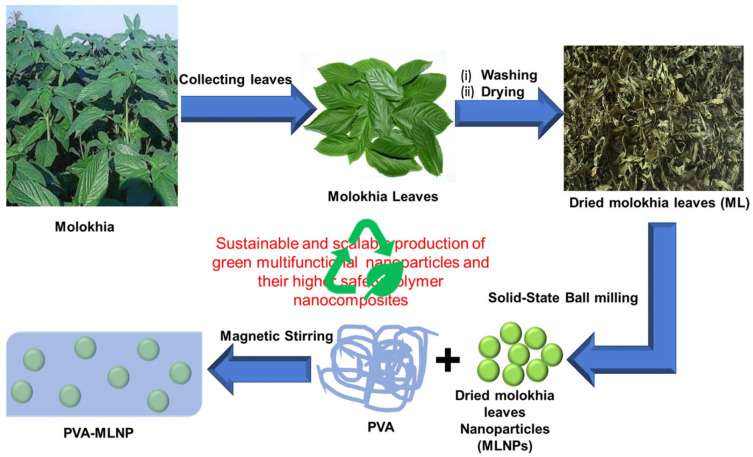
Schematic diagram representing the green synthesis process of sustainable spherical MLNPs and their well dispersed PVA nanocomposites [83].

As illustrated in Figure 3, Attia et al. [83] developed an eco-friendly and scalable approach to produce multifunctional nanoparticles from dried molokhia leaves via a one-pot solid-state ball-milling method, yielding spherical particles with an average size of 8.5 nm. When incorporated into PVA matrices, these molokhia leaf nanoparticles (MLNPs) formed nanocomposite films with enhanced fire safety, antibacterial activity, and UV protection. A key factor in the superior performance of these composites was the formation of extensive hydrogen bonds between the hydroxyl groups of PVA chains and the surface functionalities of MLNPs. These hydrogen-bonding interactions not only ensured homogeneous nanoparticle dispersion but also enabled PVA chains to wrap around MLNPs, leading to strong interfacial adhesion and stable supramolecular structures. The thermal stability of PVA and its MLNP-based nanocomposites was investigated through thermogravimetric analysis. Pure PVA exhibited two main degradation stages: an initial step beginning at 260 °C with a maximum weight loss at 315 °C, attributed to hydroxyl group release, followed by polymer chain decomposition starting at 420 °C, leaving only 1.05 wt% char at 750 °C. Incorporation of MLNPs shifted both onset and maximum degradation temperatures upward (T_onset1_ = 265 °C, T_max_ = 326 °C, T_onset2_ = 426 °C), while significantly increasing char yield to 7 wt% and reducing mass loss rate. This improvement was attributed to the MLNPs’ ability to promote protective char formation. Enhanced dispersion and nanoscale effects further strengthened this charring efficiency. Increasing MLNP loading amplified the effect, with PVA-MLNP-20 yielding 14.2 wt% char and PVA-MLNP-50 reaching 26.8 wt%, demonstrating superior thermal stability and flame retardancy. This robust hydrogen-bonding network played a central role in inducing char formation during combustion, thereby reducing the burning rate of pristine PVA from 125 mm/min to complete self-extinguishment at higher MLNP loadings. The same interactions also contributed to enhanced UV-shielding and antibacterial effects, as they stabilized the distribution of active functional groups within the matrix.

#### 3.1.2. Electrostatic Interactions

Electrostatic interactions play a significant role in the physical blending modification of PVA, particularly when it is combined with charged polymers or nanoparticles [91]. PVA itself is a neutral polymer, but its abundant hydroxyl groups allow it to interact strongly with oppositely charged species through electrostatic attraction. When blended with polyelectrolytes such as chitosan, polyacrylic acid, or sulfonated polymers, these interactions can lead to the formation of physically crosslinked networks or polyelectrolyte complexes. Such networks enhance the miscibility and interfacial adhesion between components, resulting in improved mechanical strength, dimensional stability, and resistance to phase separation. Similarly, incorporation of charged inorganic fillers, such as layered silicates, metal oxides, or functionalized nanoparticles, can exploit electrostatic interactions to achieve uniform dispersion and strong filler-matrix adhesion.

**Figure 4:**
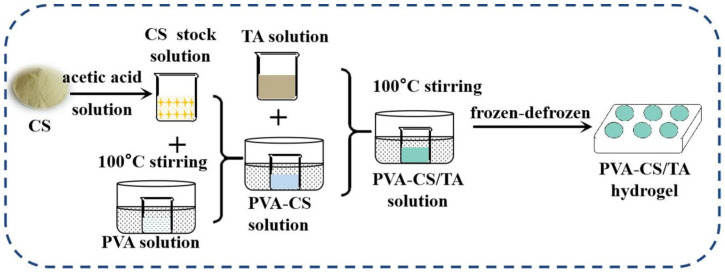
Schematic presentation of the preparation steps of PVA-CS/TA hydrogel [85].

Zhang et al. [85] reported the fabrication of biocompatible PVA-CS/TA hydrogel as illustrated in Figure 4, which shows markedly improved mechanical strength and self-healing performance due to the incorporation of chitosan. Notably, the abundant hydroxyl groups on PVA chains confer partial negative charges, enabling strong electrostatic interactions with positively charged sites on the nanofillers. With a fixed PVA content of 30 wt.% and a combined CS and TA content of 3 wt.%, increasing the CS content to 1 wt.% resulted in a tensile strength of 447 kPa and a self-healing efficiency of 84% after 2 h. This represents a significant enhancement compared with previously reported biocompatible self-healing hydrogels, which generally exhibit tensile strengths below 300 kPa, highlighting the effectiveness of chitosan in reinforcing the hydrogel through reversible hydrogen bonding and stronger electrostatic interactions.

#### 3.1.3. Van Der Waals Interactions

Van der Waals interactions represent another important mechanism in the physical blending modification of PVA. These weak, non-covalent forces arise from transient dipol-dipole or induced dipole interactions between PVA chains and blended polymers, small molecules, or nanofillers. Although individually weak, van der Waals interactions can collectively enhance compatibility and interfacial adhesion in PVA composites, particularly when the contact area between components is large, as in nanocomposites. For example, blending PVA with hydrophobic polymers, carbon-based nanomaterials, or layered silicates allows van der Waals forces to contribute to uniform dispersion, reduce phase separation, and stabilize the composite structure without the need for chemical bonding. These interactions can also influence the packing density and chain mobility of PVA, affecting crystallinity, mechanical strength, barrier performance, and thermal stability.

Nguyen et al. [86] investigated the enhancement of flame retardancy in PVA-based composite films reinforced with nanoclay and multi-walled carbon nanotubes (MWCNTs). Using solution blending and casting, PVA films were prepared with nanoclay. The optimized composite containing 3% nanoclay and 0.5% MWCNTs exhibited the highest mechanical performance, with tensile strength increasing by 40.8% from 32.8 to 46.2 MPa and flexural strength rising by 34.5% from 53.2 to 71.5 MPa. Importantly, the limiting oxygen index (LOI) of the optimized composite reached 31.5%, compared to 19.8% for pure PVA, and the material achieved a UL-94 V-0 rating, confirming its self-extinguishing ability.

Although physical blending is simple and cost-effective, it faces several intrinsic challenges. High loadings of nanofillers such as clays or carbon nanotubes can lead to aggregation, creating defect sites that compromise mechanical properties and optical transparency. Most blended flame retardants are not covalently bonded to the PVA matrix, which may result in leaching or migration over time, particularly in aqueous or humid conditions, reducing flame-retardant efficiency and potentially causing environmental concerns. Additionally, while some fillers can reinforce the matrix, many act as stress concentrators at high concentrations, decreasing flexibility and tensile strength. Achieving uniform dispersion often requires organic modifiers or surfactants, which can complicate processing and reduce the biocompatibility and sustainability of the composite.

### 3.2. Chemical Modification of PVA

Chemical modification of PVA provides an effective means to tailor its properties for enhanced performance in areas such as flame retardancy, mechanical strength, and biocompatibility [92]. The abundant hydroxyl groups along the PVA backbone allow for diverse reactions, including esterification, etherification, acetalization, and graft copolymerization, facilitating the introduction of functional groups that enhance hydrophobicity, thermal stability, and chemical resistance. Additionally, cross-linking with agents like glutaraldehyde or borates can significantly improve mechanical strength and dimensional stability.

#### 3.2.1. Esterification

Esterification is a common chemical modification of PVA that involves the reaction between the hydroxyl groups of PVA and the carboxyl groups of carboxylic acids, acid anhydrides, or acid chlorides to form ester linkages [93]. This reaction can develop an ester-bridged network within the polymer matrix, improving structural cohesion and stability. By converting some of the hydrophilic hydroxyl groups into less polar ester groups, esterification reduces the overall hydrophilicity of PVA and enhances its water resistance. Additionally, the introduced ester groups can serve as reactive sites for further chemical modifications or improve compatibility with other polymers and additives, enabling the design of multifunctional PVA-based materials.

Reina et al. [94] demonstrated that esterification of PVA with 1-oxo-2,6,7-trioxa-1-phosphabicyclo [2.2.2]octane effectively enhanced flame retardancy by covalently introducing phosphorus moieties. The modified polymers showed an onset decomposition temperature about 40 °C higher than pristine PVA, with char residues exceeding 30 wt% at 700 °C, nearly three times greater than the unmodified polymer. Cone calorimetry revealed a reduction of more than 50% in peak heat release rate, confirming that esterification promotes stable barrier formation and imparts superior flame retardant efficiency compared with unmodified PVA.

#### 3.2.2. Acetalization or Ketalization

Acetalization or ketalization is a widely used chemical modification strategy for PVA, taking advantage of its abundant hydroxyl groups to form stable cyclic or acyclic linkages with aldehydes or ketones [95,96]. In acetalization, the hydroxyl groups of PVA react with aldehydes, such as formaldehyde or glutaraldehyde, to generate acetal bonds, while in ketalization, they react with ketones under acidic conditions to form ketal linkages. These reactions create a crosslinked polymer network that significantly improves water resistance, chemical stability, and mechanical strength, since the acetal or ketal groups reduce the number of free hydroxyl groups and limit chain mobility.

Ten bio-derived ketones, four fossil-derived ketones, and one bio-based aldehyde were employed for PVA attachment, including fermentation-derived acetone and its derivatives, naturally occurring ketones, and platform chemicals, alongside fossil-based solvents such as butanone, 3-pentanone, cyclopentanone, and cyclohexanone. Miller et al. [97] demonstrated that acetalization and ketalization of PVA with various aldehydes and biobased ketones produced polyvinyl ketals with tunable properties. The reactions achieved ketalization levels of 15.3–69.2% and increased molecular weights to 24,400–41,100 Da. Thermal stability improved markedly, with glass transition temperatures rising from 75 °C in unmodified PVA to 78–127 °C in ketalized derivatives and up to 138 °C with furfural acetalization. Kinetic studies showed rapid equilibrium at mild conditions, while hydrolytic degradation varied with environment, ranging from complete hydrolysis in 1 day at acidic pH to long-term stability under neutral conditions.

#### 3.2.3. Cross-Linking Strategy

PVA can be structurally reinforced through chemical or physical cross-linking, where its abundant hydroxyl groups react with agents such as glutaraldehyde, boric acid, epoxides, phenyl dichlorophosphate, or divinyl compounds to form covalent or coordination bonds within the polymer network [98]. This cross-linking strategy effectively restricts chain mobility, leading to improvements in mechanical strength, dimensional stability, thermal resistance, and water durability. In addition, the incorporation of functional moieties or nanofillers during the cross-linking process allows for the development of multifunctional PVA-based materials with enhanced properties such as ionic conductivity, flame retardancy, and biocompatibility, broadening their potential applications in membranes, biomedical devices, coatings, and environmentally friendly composites.

**Figure 5:**
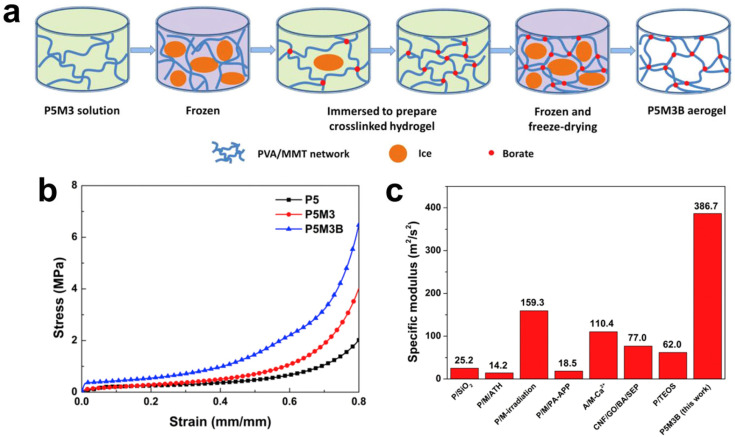
(**a**) Preparation of the crosslinked aerogels. (**b**) Compressive stress–strain curves of P5, P5M3 and P5M3B aerogels. (**c**) A summary of specific moduli for present fire safety aerogels [100]. Copyright 2017. Reproduced with permission from Elsevier Science Ltd.

Tăchiță et al. [99] demonstrated that the chemical phosphorylation of PVA significantly improved its flame-retardant performance. Phosphorus-modified PVAs, synthesized from PVAs of three different molecular weights, exhibited glass transition temperatures ranging from 58.49 to 67.65 °C, reflecting enhanced thermal stability. Thermogravimetric analysis showed an increased char yield, while micro-scale cone calorimetry revealed reduced heat release rates. Liao et al. [100] reported a robust and flame-retardant borate-crosslinked poly(vinyl alcohol)/montmorillonite aerogel prepared by immersing frozen PVA/MMT sol into a borax solution, where borate crosslinked with PVA diols during ice melting (Figure 5a). Freeze-drying produced a compact network with optimized crosslinking, showing superior mechanical strength at low density. The borate-crosslinked structure provided excellent flame retardancy, achieving a limiting oxygen index of 27.6%, UL-94 V-0 rating, and significantly suppressed combustion characteristics. Figure 5b,c show that PVA/clay composite aerogels outperform pure PVA, with P5M3 exhibiting a 2.7-fold higher specific modulus and melt-crosslinked P5M3B reaching nearly a 10-fold enhancement. In P5M3, the decomposition peak at ~70 °C arises from crystalline water release, whereas in the borate-crosslinked P5M3B aerogel these peak shifts to ~80 °C due to reversible PVA–borate interactions. Borate crosslinking increases T_d_,_max_, lowers the weight loss rate, and, together with MMT, enhances residue formation through barrier and migration resistance effects. The catalytic role of boron further promotes stable char generation. As a result, P5M3B outperforms previously reported PVA/clay and other fire-safe aerogels, highlighting the effectiveness of borate and melt crosslinking in producing lightweight yet thermally robust structures. Liu et al. [88] developed a multi-crosslinked PVA composite using a hexachlorocyclotriphosphazene derivative and chitosan, followed by copper ion chelation. This structure greatly improved flame retardancy, reducing peak heat release rate (PHRR) and total heat release (THR) by 52.38% and 24.22%, increasing LOI and char formation, and decreasing total smoke production by 91.00%. The composite also exhibited strong antibacterial activity, with inhibition rates of 99.67% against *E. coli* and 92.45% against *S. aureus*.

Chemical modification provides permanent and uniform flame retardancy in PVA, but it also presents several limitations. The required synthesis often involves complex conditions such as controlled temperatures, inert atmospheres, catalysts, and organic solvents, raising concerns about scalability, cost, and environmental impact compared with simple blending. Reactions like esterification or crosslinking can disrupt the crystallinity and hydrogen-bonding network of PVA, potentially reducing tensile strength and increasing brittleness. Harsh conditions may also cause chain scission or degradation, lower molecular weight and compromising structural integrity.

### 3.3. Surface Engineering of PVA

#### 3.3.1. Plasma Treatment

Plasma treatment is a versatile and effective surface modification technique widely used to enhance the properties of PVA without altering its bulk characteristics [101]. By exposing PVA surfaces to plasma, functional groups such as hydroxyl, carboxyl, and carbonyl are introduced, increasing surface energy and improving hydrophilicity, adhesion, and reactivity. When combined with polydopamine (PDA), a mussel-inspired bio-polymer known for its exceptional adhesive properties and structural stability, plasma-treated PVA can achieve stronger interfacial bonding, improved coating uniformity, and enhanced durability in composite or coating applications.

**Figure 6:**
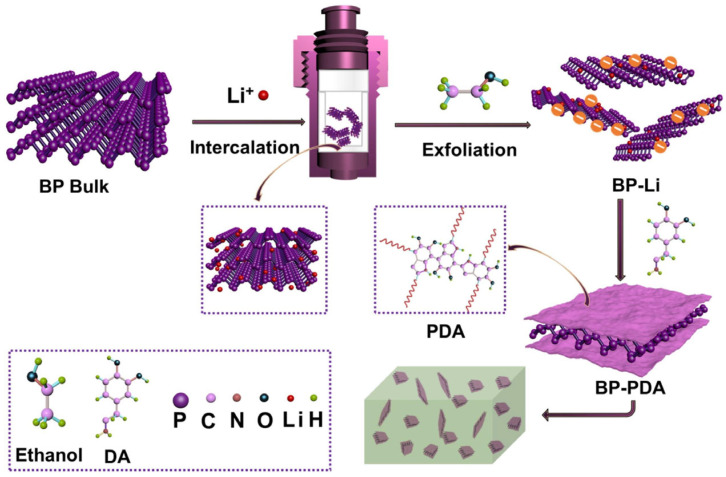
Schematic diagram for synthesis of BP-PDA hybrid and fabrication of PVA/BP-PDA nanocomposites [89]. Copyright 2020. Reproduced with permission from Elsevier Science Ltd.

Hu et al. [89] demonstrated that plasma treatment significantly enhances the performance of PVA nanocomposites containing black phosphorus (BP) nanosheets. As illustrated in Figure 6, plasma treatment of PVA enhanced the adhesion and dispersion of BP-PDA, which significantly improved its mechanical and flame-retardant performance, achieving a 57.2% reduction in peak heat release rate and an 81.1% increase in tensile strength. The combination of PDA encapsulation, polymer embedding and plasma surface activation also improved the long-term air stability of BP, highlighting plasma treatment as an effective strategy for producing high-performance polymer nanocomposites.

#### 3.3.2. Layer-by-Layer (LbL) Assembly

**Figure 7:**
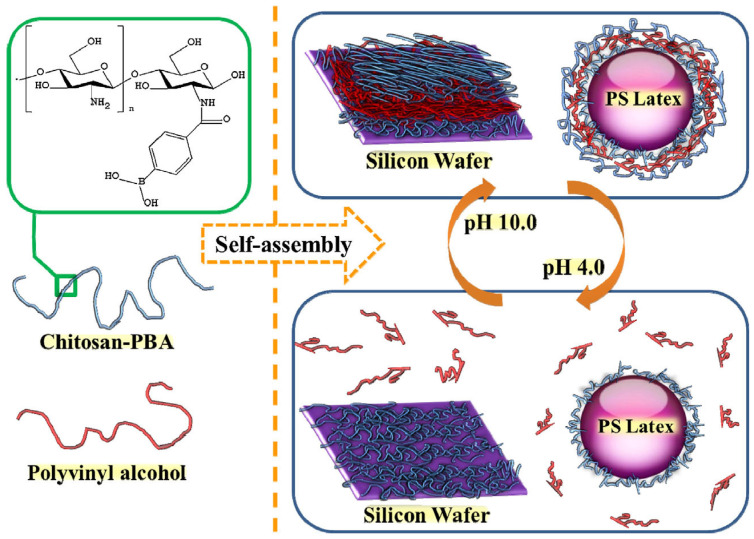
Schematic illustration of pH-dependent self-assembly of chitosan–PBA and PVA. The supporting substrate is either silicon wafer or monodispersed polystyrene latexes with 500 nm diameter [102]. Copyright 2016. Reproduced with permission from Elsevier Science Ltd.

**Figure 8:**
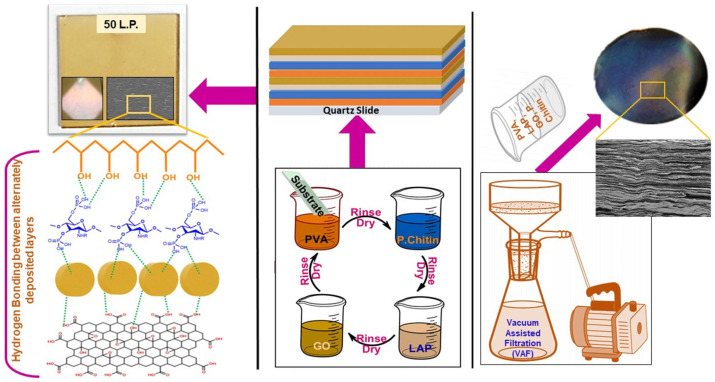
Schematic of LbL and VAF assemblies for the deposition of nacre-like multilayered films (SEM of cross section and photograph of films shown on top left and top right) [103]. Copyright 2022. Reproduced with permission from Elsevier Science Ltd.

LbL assembly is an effective technique for engineering the surface of PVA by sequentially depositing alternating layers of oppositely charged polymers, nanoparticles, or other functional materials. This approach relies on electrostatic interactions, hydrogen bonding, or other reversible forces to build well-defined, uniform coatings on the PVA surface. By controlling the number of layers, composition, and deposition conditions, LbL assembly can precisely tune surface properties such as mechanical strength, barrier performance, hydrophilicity, and flame retardancy. As depicted in Figure 7, the schematic illustrates the self-assembly process of chitosan–PBA and PVA. Chitosan–PBA, a phenylboronic acid-derivatized chitosan, was synthesized through an EDC/NHS coupling reaction, resulting in the formation of covalent amide bonds [102]. This modified chitosan together with PVA was then utilized to construct self-assembled multilayers on either smooth silicon wafers or monodispersed polystyrene latex particles with a diameter of 500 nm. At pH 10.0, multilayers of chitosan–PBA and PVA were successfully fabricated using the LBL method due to the formation of borate esters between the diols of PVA and the phenylboronic acid moieties of chitosan–PBA, while no self-assembly occurred at pH 4.0. Gill et al. [103] demonstrated that LbL assembly can produce nacre-mimetic coatings with enhanced mechanical strength and flame retardancy. As shown in Figure 8, Phosphorylated chitin was combined with PVA, graphene oxide, and laponite to form multilayered films via LbL and vacuum-assisted filtration. SEM confirmed a well-defined layered structure, and the films exhibited a pearlescent sheen, a reduced modulus of 25.53 GPa, hardness of 1.45 GPa, alongside iridescence and effective flame retardancy.

#### 3.3.3. Surface Graft Method

Surface grafting is an effective method to functionalize PVA by covalently attaching functional molecules or polymer chains onto its surface. This technique typically involves initiating chemical reactions at the hydroxyl-rich surface of PVA, such as free radical polymerization, click chemistry, or other grafting strategies, to introduce desired functionalities without affecting the bulk properties of the polymer. Surface grafting can enhance properties of PVA, including hydrophobicity, flame retardancy, chemical resistance, ionic conductivity, and biocompatibility. Liu et al. [90] reported a surface-grafting strategy to prepare high-performance flame-retardant PVA membranes, overcoming the limitations of conventional physical mixing of flame retardants. Using DPP solution coating and hydroxyl group condensation, the flame retardant was chemically grafted onto the PVA membrane, resulting in a more uniform distribution and continuous char formation during combustion. Compared to physically mixed PVA/DPP membranes, the surface-grafted PVA-DPP exhibited superior flame retardancy, enhanced thermal stability, higher melting point, improved mechanical properties, and maintained transparency. Enhanced hydrogen-bond interactions minimized defects and stress concentration, highlighting its potential for advanced packaging and high-performance applications.

Surface engineering offers an effective strategy to functionalize PVA while preserving its bulk properties, but it also has notable limitations. The durability and abrasion resistance of surface coatings are major concerns, as thin layers deposited by methods such as LBL assembly or grafting can be damaged or delaminated under mechanical stress, reducing flame-retardant performance. Plasma treatments may provide only superficial effects that are not permanent due to molecular reorientation over time. Additionally, surface modifications protect only the exterior, leaving the underlying bulk PVA highly flammable if the surface layer is breached or exposed to intense or prolonged fire.

## 4. Various Applications of PVA

### 4.1. Optical Applications

**Figure 9:**
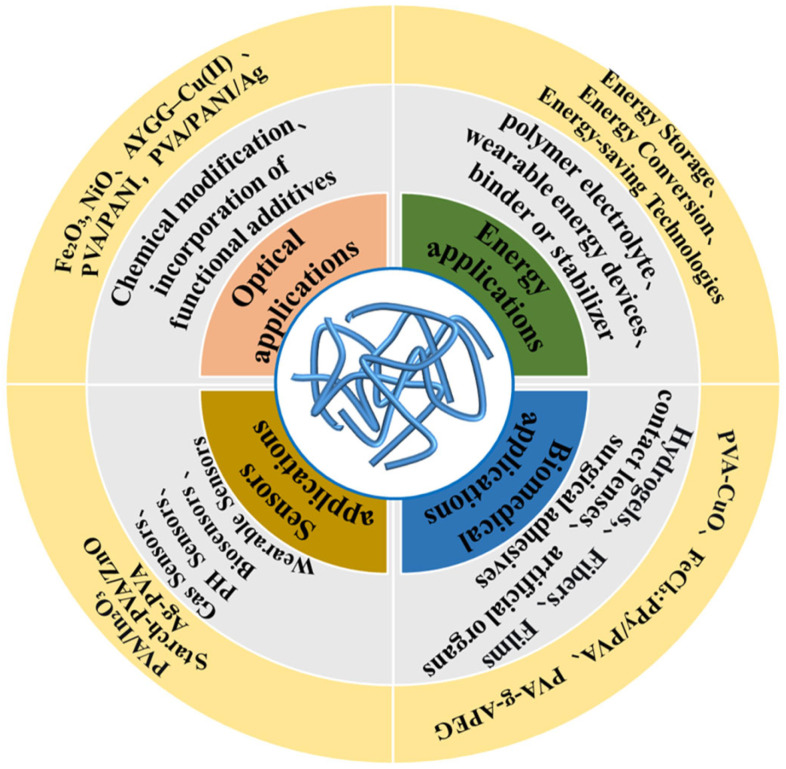
Multifunctional applications of flame-retardant PVA-based systems, including optical, energy, biomedical, and sensing fields.

PVA has emerged as a valuable material for optical applications due to its excellent film-forming ability, optical transparency, and chemical stability. Figure 9 presents a comprehensive overview of the multifunctional applications of flame-retardant PVA-based systems. PVA can be processed into uniform, smooth films with high light transmittance, making it suitable for lenses, optical coatings, and protective layers in electronic displays. Its abundant hydroxyl groups allow for chemical modification or incorporation of functional additives such as dyes, nanoparticles, or photoactive compounds, enabling tunable refractive indices, light absorption, and emission properties. Rashad et al. [104] investigated the structural and optical properties of pure and nanoparticle-doped PVA, prepared via a casting method with 1 wt% Fe_2_O_3_ or NiO. Nonlinear optical susceptibility and refractive index were also doping-dependent, with Fe_2_O_3_ doping notably enhancing optical conductivity. These results indicate the strong potential of pure and doped PVA for optoelectronic applications. Khalil et al. [105] developed AYGG–Cu(II) doped PVA films with spherical, amorphous particles. The Cu(II) ions coordinated with AYGG through phenolic hydroxyl and azo nitrogen groups. The films showed high dispersion for ultraviolet and high-frequency visible light, making them suitable as selective optical filters for applications such as solar cell protection and packaging.

Polyaniline, as a polymer, was also used to enhance the optical properties of PVA. Atta et al. [106] successfully synthesized flexible PVA/PANI and PVA/PANI/Ag nanocomposite films via solution casting and reduction methods, embedding uniform silver nanoparticles (AgNPs) with sizes ranging from 12 to 36 nm. The incorporation of PANI and AgNPs significantly modified the optical properties of PVA. The optical bandgap and absorption edge of the films decreased compared to pristine PVA, while the Urbach energy, band tail, and the number of carbon clusters increased with higher PANI and Ag contents. Additionally, the refractive index and optical dielectric constant declined as the PANI and AgNPs loading increased, whereas the optical conductivity of the films improved due to an enhanced density of localized states in the band structure. The refractive index and related dispersion parameters were further analyzed using the Wemple–DiDomenico model, confirming that these nanocomposite films exhibit tunable optical behavior suitable for advanced optoelectronic applications.

### 4.2. Energy Applications

PVA has found wide-ranging applications in the energy field due to its film-forming capability, chemical stability, flexibility, and ability to interact with various functional materials. PVA is commonly used as a polymer electrolyte in fuel cells, lithium-ion batteries, and supercapacitors, where it provides ionic conductivity and mechanical stability. Its hydroxyl-rich structure allows for easy incorporation of salts, acids, or nanoparticles, enhancing proton conductivity and overall electrochemical performance. PVA-based hydrogels and membranes are also employed in flexible and wearable energy devices, offering both mechanical robustness and efficient ion transport. Additionally, PVA serves as a binder or stabilizer in energy storage and conversion systems, contributing to improved durability and interface compatibility. Saikia et al. [107] prepared PVA thin films on glass substrates, highlighting their suitability for solar cells and various optoelectronic devices, such as semiconductor lasers, nonlinear optical components, thin-film transistors, LEDs, and photodetectors. Mohanapriya et al. [108] fabricated PVA/polystyrene sulfonic acid (PSSA)/carbon black nanoparticle (CBNP) thin films with different CBNP loadings, targeting flexible energy storage applications.

Zr^4+^, as a dopant, was used to enhance the mechanical and energy-harvesting properties of PVA fiber films. Yin et al. [109] prepared biodegradable CPZ/PVA hydrogel fiber films via electrospinning, where Zr^4+^ coordinated with functional groups to develop a soft/hard chain interpenetrating structure, significantly improving mechanical performance. The incorporation of Zr^4+^ increased the maximum tensile strength by 2.16 times and the maximum elongation at break by 3.44 times compared with pristine PVA fibers. This structural modification also redistributed polymer electron clouds, enhancing surface charge density and enabling the films to function effectively as biodegradable triboelectric nanogenerators (BD-TENG) with a power density of up to 225 μW cm^−2^ over 8000 cycles. In passive daytime radiative cooling (PDRC) applications, the films exhibited a solar reflectivity of 94.23% and mid-infrared emissivity of 67.45%, resulting in a surface temperature reduction of 26.2 °C. These results demonstrate that Zr^4+^-doped CPZ/PVA fiber films combine enhanced mechanical strength, biodegradability, and energy conversion capabilities, offering promising applications in green energy and energy-saving technologies.

### 4.3. Sensors Applications

PVA has emerged as a highly versatile material for sensor applications owing to its remarkable film-forming capability, mechanical flexibility, chemical stability, and rich hydroxyl functionality, which facilitates facile modification with a wide range of sensing moieties. PVA-based composites and hydrogel systems have been extensively engineered for gas sensing applications, where the integration of conductive, catalytic, or reactive nanoparticles enhances sensitivity and selectivity toward gases such as ammonia, nitrogen dioxide, and various volatile organic compounds. In pH sensing, PVA provides a robust and chemically stable matrix for immobilizing pH-responsive dyes, polymers, or nanomaterials, enabling rapid, reversible, and accurate detection of acidic or basic environments. Beyond gas and pH detection, PVA serves as an effective platform for chemical sensors targeting metal ions, organic analytes, and biomolecules through selective binding, chelation, or enzyme-mediated recognition. The inherent mechanical flexibility of PVA, coupled with its hydrogel-forming ability, further allows its application in pressure and strain sensors, as well as in wearable and deformable electronics, where mechanical deformation can be efficiently transduced into measurable electrical signals. Moreover, the tunable porosity, swelling behavior, and biocompatibility of PVA expand its utility in multifunctional sensing devices, including environmental monitoring systems, healthcare diagnostics, and next-generation smart electronic technologies. Consequently, PVA stands out as a highly adaptable material for developing innovative, high-performance sensor platforms with broad applicability across diverse fields.

Lim et al. [110] prepared a gas sensor using PVA/In_2_O_3_ nanocomposites, which detected H_2_ gas by monitoring changes in thin-film surface conductivity. Bittencourt et al. [111] created a gas sensor based on PVA and polyaniline (PVA/PANI) nanofibers, measuring gas mass through variations in electrical current during exposure to ammonia and nitrogen gases. Jayakumar et al. [112] developed starch-PVA thin films embedded with ZnO nanoparticles as pH sensors, where color changes in the films used as food packaging indicated pH variations. Ramesh et al. [113] fabricated silver nanoparticle-embedded PVA (Ag-PVA) thin films as selective sensors for Hg^2+^, Hg_2_^2+^, and elemental mercury in aqueous solutions, capable of detecting mercury ion concentrations ranging from 10 ppb to 1 ppm.

### 4.4. Biomedical Applications

PVA is extensively utilized in biomedical applications owing to its excellent biocompatibility, non-toxicity, chemical stability, and film-forming ability. PVA can form hydrogels, fibers, and films that are used in wound dressings, drug delivery systems, and tissue engineering scaffolds, providing a moist and supportive environment for cell growth and tissue repair. Its abundant hydroxyl groups allow for chemical modification or crosslinking to tailor mechanical properties, degradation rates, and bioactivity [114]. PVA-based materials are also employed in contact lenses, artificial organs, and surgical adhesives due to their flexibility, transparency, and compatibility with biological tissues. Dhiman et al. [115] prepared PVA thin films embedded with copper oxide (PVA-CuO) on indium tin oxide (ITO)-coated glass for glucose monitoring and blood sugar measurement. Shi et al. [116] developed thin films of FeCl_3_ incorporated into polypyrrole-polyvinyl alcohol (FeCl_3_:PPy/PVA) to function as strain sensors capable of detecting various physiological signals.

PVA has shown significant promise in biomedical applications, particularly in the development of advanced medical dressings. Guo et al. [117] reported the fabrication of PVA/polyethylene oxide (PEO)/PVA-g-APEG nanofiber membranes via electrospinning, using polyvinyl alcohol-allyl polyethylene glycol graft copolymer (PVA-g-APEG) as a compatibilizer to enhance structural and functional properties. The incorporation of 4 wt% PVA-g-APEG resulted in the narrowest nanofiber diameter distribution, with an average fiber diameter of 267.18 nm and a maximum porosity of 77.90%, closely mimicking the natural extracellular matrix. Additionally, the nanofiber membranes exhibited excellent lipophilicity, rapid liquid absorption, and enhanced swelling capacity, providing a stable and moist environment for wound healing. Biocompatibility assays demonstrated a cell proliferation rate exceeding 100% and a cytotoxicity grade of 0, while the membranes effectively avoided adhesion with cells. These quantitative improvements underscore the potential of PVA/PEO/PVA-g-APEG nanofiber membranes as high-performance, safe, and effective materials for burn and wound medical dressings, highlighting their practical applicability in regenerative medicine.

## 5. Concluding Remarks and Future Aspects

PVA is a versatile polymer with a linear backbone rich in hydroxyl groups, which allows extensive hydrogen bonding. This feature provides excellent water solubility, strong mechanical performance, good film-forming ability, effective barrier properties, and biocompatibility. These inherent characteristics enable a wide range of applications, including adhesives, packaging, textiles, construction materials, biomedical devices, and optoelectronic components. Despite these advantages, PVA exhibits high flammability, with rapid thermal degradation and the release of combustible volatiles, limiting its use in environments requiring fire safety.

Considerable progress has been made in improving the fire resistance of PVA through physical blending, chemical modification, and surface engineering. Hydrogen bonding, electrostatic forces, and van der Waals interactions in physical blends promote uniform dispersion of fillers, strong interfacial adhesion, and enhanced char formation, resulting in improved flame retardancy while maintaining mechanical and barrier performance. Chemical modifications such as esterification, acetalization, ketalization, phosphorylation, and cross-linking allow precise control of structural and thermal stability. These modifications reduce flammability and improve water resistance, mechanical strength, and multifunctionality. Surface engineering techniques including plasma treatment, LBL assembly, and surface grafting provide localized functionalization, enhancing flame-retardant, barrier, and optical properties without altering the bulk polymer. Together, these strategies form a comprehensive approach for producing high-performance, flame-retardant PVA materials.

Future developments should focus on integrating sustainable and environmentally friendly strategies into PVA material design. The use of bio-based additives, naturally derived nanoparticles, and green cross-linkers can minimize ecological impact while improving fire safety. Multifunctional designs that combine flame retardancy with optical, antimicrobial, energy storage, or sensing capabilities offer opportunities for applications in flexible electronics, smart packaging, and biomedical devices. Advanced molecular design, computational modeling, and precise characterization techniques can further reveal the relationship between structure and properties, enabling rational design of highly efficient, multifunctional materials.

The continued development of flame-retardant and multifunctional PVA materials demonstrates the polymer’s potential to satisfy strict safety and performance standards. By combining chemical, physical, and surface engineering methods with sustainable materials, future PVA materials can achieve high performance, multifunctionality, and environmental compatibility, expanding their use across diverse industrial and technological applications.

## Figures and Tables

**Table 1:** Comparison of material information and flame-retardant properties of various material systems.

Composition of Materials	Preparation Method	Fillers Content (wt%)	LOI (%)	PHRR (%)	THR (%)	UL-94rating	Ref.
TE/PVA	Hydrogen bond association	10	↑ 25.5	−43	−42	V-0	[73]
GP/PVA	Hydrogen bond association	15	↑ 31.2	−73	−27	V-0	[82]
MLNPs/PVA	Hydrogen bond association	50	—	0	—	—	[83]
4N-2456/PVA	Hydrogen bond association	0.5	—	—	—	—	[84]
CS/TA/PVA	Electrostatic interactions	CS: 0–1.5 TA: 1.5–3	—	—	—	—	[85]
Nanoclay/MWCNTs/PVA	Van der Waals interactions	Nanoclay: 3 MWCNTs: 0.5	↑ 31.5	—	—	V-0	[86]
GO/PA/PVA	Esterification	—	↑ 36	−88.6	−66.5	V-0	[62]
MCNFs/PVA	Layer-by-Layer (LbL) assembly	6	—	−37	−15	—	[87]
SHCP@CS-Cu/PVA	Cross-linking strategy	20	↑ 29.1	−52.38	−13.91	—	[88]
BP-PDA/PVA	Plasma treatment	5	—	−57.2	−47.9	—	[89]
DPP/PVA	Surface graft method	7.44	—	—	—	V-0	[90]

## Data Availability

Not applicable.
